# Review of the target trial methodological approach on treatment effect estimates in kidney failure: protocol for a systematic assessment

**DOI:** 10.1186/s13643-024-02672-4

**Published:** 2024-11-14

**Authors:** Jule Pinter, David J. Tunnicliffe, Pooshwikaa Karunikaikumar, Anastasios Anastasiadis, Robert K. Hills

**Affiliations:** 1 University Hospital Würzburg, 97080 Würzburg, Germany; 2 Sydney School of Public Health, The University of Sydney Centre for Kidney Research, The Children’s Hospital at Westmead, Camperdown, Australia; 3 Julius Maximillian University, Würzburg, Germany; 4 Nuffield Department of Population Health, University of Oxford, Oxford, UK

**Keywords:** Methodological systematic review, Target trial emulation, Kidney failure

## Abstract

**Background:**

Patients with kidney failure often lack robust evidence because they are excluded from randomized trials. Trial emulation provides an alternative approach to derive treatment effect estimates when randomized trials cannot be conducted. Critical questions about the comparative efficacy and safety of interventions in kidney failure are now being answered using this approach or parts of it. However, variations and inconsistencies in reporting cast doubt on the reliability and validity of effect estimates not derived from randomized trials. The aim of this methodological systematic review is to understand the extent to which the target study approach is used in kidney failure and the appropriateness of this approach. By identifying and evaluating studies that qualify as emulating a target trial, compared with studies that did not apply the principles. We aim to provide more specific methodological guidance to increase the clarity and reliability of reporting treatment effect estimates when running a trial in kidney failure is not feasible.

**Methods:**

This protocol is developed in accordance with Preferred Reporting Items for Systematic reviews and Meta-Analyses Protocols (PRISMA-P) statement. MEDLINE, Embase, and reference lists (backwards citation chasing) will be searched up until 1st July 2023 and the search updated prior to publication to identify all studies evaluating patient outcomes in late-stage kidney disease and failure that use target trial emulation as the primary approach for analysis. Two authors (A. A., P. K.) will select articles based on title and abstract and then full text, with a third reviewer settling disagreements (J. P.). The prespecified variables will be extracted, and the risk of bias will be assessed by at least two authors (A. A., P. K., A. N.) using prespecified data forms. This will enable the determination of the robustness of the methodological quality of observational studies in using the whole or elements of the target trial approach. We will thereby assess their ability to reliably report treatment effect estimates.

**Discussion:**

We will provide specific methodological recommendations on how to design target trials and model assumptions for emulation to get reliable treatment effect estimates for therapeutic interventions in kidney failure.

**Methodological systematic review registration:**

Open Science Framework: Identifier https://doi.org/10.17605/OSF.IO/Z4Y29.

**Supplementary Information:**

The online version contains supplementary material available at 10.1186/s13643-024-02672-4.

## Introduction

### Background and rationale

The lack of effective treatments and stagnant mortality rates in patients with kidney failure have created a gap between the urgent need for improved patient outcomes and the limited reliable evidence available to inform clinical decisions. Nephrology ranks lowest among internal medicine specialties in terms of conducting and delivering randomized controlled trials (RCTs). This ranking is based on comparative analyses of trial output and funding across different fields of internal medicine, with nephrology consistently showing fewer trials and lower research funding over the past two decades [1–3]. Vulnerable patient cohorts such as those with kidney failure have often been excluded from cardiovascular RCTs [1, 4].

Although the number and quality of trials for patients with chronic kidney disease have recently increased, many research questions regarding comparative effectiveness and safety of patient outcomes, particularly in dialysis, remain unaddressed. The mounting pressures of increased bureaucracy, cost explosions, and the need for highly organized, large-scale infrastructure have made it increasingly difficult to conduct timely, cost-effective, and pragmatic trials in advanced kidney disease. The low-certainty evidence in management of kidney failure makes treatment decisions for improved patient outcomes difficult. Innovative solutions to improve evidence are needed to address the unmet need. If the execution of clinical trials is unfeasible, too expensive, or not timely, target trial emulation could be an alternative, second-best option to provide answers regarding the comparative effectiveness, safety, and use of drugs and therapeutic interventions to improve patient outcomes in kidney failure.

Target trial emulation is an advanced and well-established epidemiological framework to assess questions of causal inference in non-randomized studies [5]. With the design of a target trial addressing a causal research question, real-world evidence is utilized to emulate a hypothetical trial scenario. It is a technique to minimize inadvertent biases (e.g., immortal or lead time or survivor bias) that are often created by investigators in non-randomized studies. As such, results of target trial emulation have repeatedly been shown to resemble more closely trustworthy and reliable results than traditional observational studies [6–11].

The concept has recently been introduced to nephrology and warrants systematic assessment [12]. Through a methodological systematic review, we aim to understand the validity and utility of the target trial approach to answering causal questions in the most vulnerable subset of patients with kidney failure when traditional randomized trials are unfeasible, unethical, or untimely.

### Objectives

This systematic review aims to investigate the use, reporting scope, and risk of bias associated with target trial emulation in patients with kidney failure. Specifically, it will assess the extent of target trial application in kidney failure, critically appraise the reliability and trustworthiness of treatment effect estimates achieved through the target trial framework methodology, identify reasons for failure, and provide recommendations for future applications to avoid biases.

Target trial emulation is a sophisticated technique for testing causal inference in non-randomized studies by systematically eliminating inadvertent and unnecessary biases. However, it can be challenging to conduct, depending on the clinical importance of the research question, the design and specification of the target trial, the quality of the data source and emulation, and the robustness of the statistical methods used. In kidney failure studies, high comorbidity, complex disease courses, and short survival times present additional challenges. This review aims to understand the limitations of implementing target emulation studies, the questions being addressed, and their outcomes in kidney failure research.

## Methods

The protocol follows PRISMA-P guidelines for reporting (Supplementary Material 1) [13]. We will report the review according to the Preferred Reporting Items for Systematic reviews and Meta-Analyses (PRISMA) [14].

### Eligibility criteria

To assess the methodology of target trial emulation systematically, studies will be included irrespective of data source, setting, or country. Clinical questions were separated according to Table 1 summarizing the PICOS (population, intervention, comparison, outcome, study design) framework applied in this review.

**Table 1:** PICOS table

Criteria framework	Inclusion criteria	Exclusion criteria
**Population**	Adults and children with late-stage chronic kidney disease and kidney failure being defined as chronic kidney disease stages IV and V with and without renal replacement therapy as defined by KDIGO [15]	Adults and children without late-stage chronic kidney disease and kidney failure
**Interventions**	Study-specific interventions evaluating comparative effectiveness, safety, and use of pharmaceutical drugs and or therapeutic interventions such as timing of renal replacement therapy start, choice of modality, transplantation, or other forms of surgery	Nontherapeutic interventions, such as lifestyle and educational interventions or exposure-related risk factors
**Comparison**	Study-specific comparison evaluating comparative effectiveness, safety, and use of drugs and or therapeutic interventions	
**Outcomes**	Major objective clinical endpoints, such as all-cause or cause-specific mortality or major adverse cardiovascular events and or composite outcomes using study-specific definitions, such as kidney composites	Nonclinical endpoints, such as surrogate outcomes of kidney function — i.e., estimated glomerular filtration rate, serum creatinine
**Study design**	Observational studies investigating the effects of interventions. Full text published in English or German	RCTs, systematic reviews, and non-primary research. Non-English and non-German studies

#### Population

We will include studies that include adults and children with kidney failure being defined as late-stage chronic kidney disease and end-stage kidney failure (stage IV or higher as per KDIGO) [15] with and without renal replacement therapy (Supplementary Material 2 for kidney disease nomenclature).

Recent systematic reviews on the quantity and reporting of kidney research have shown that patients with who have or are at risk of progression to kidney failure are most often excluded from trials [1, 2]. Due to the limited number of trials in this population, the target trial approach seems most relevant and applicable. We will therefore include this most vulnerable patient cohort and leave out patient cohorts with early and acute disease settings which are more often represented in trials [3]. A list of complete exclusion criteria is provided in Supplementary Material 3.

#### Intervention and comparator

Target trial emulation derives plausible treatment effect estimates from specific interventions. We will therefore focus on all causal research question focusing on therapeutic interventions that evaluate efficacy, comparative effectiveness, or safety. This will include drug comparisons and specific interventions, such as initiation of renal replacement therapy or transplantation. Observational studies focusing on noncausal research questions, such as exposure-related risk estimates, will therefore be excluded.

#### Main outcomes

Relevant patient outcomes are major objective clinical endpoints typically used in published outcome-driven trials [16–18] and core outcome definitions [19] (Table 1). Nonrelevant surrogate outcomes that do not allow establishing cause and effect on clinical outcomes will therefore be excluded.

### Study design

Observational studies that investigate the effects of interventions are eligible that use the whole or parts of the target trial emulation approach. RCTs, systematic reviews, and non-primary research (i.e., case series/editorials/reports/narrative reviews) are outside of scope.

We will only assess studies published in English and German which we can assess on native speaker level due to limited number of resources.

### Information sources, search strategy, and study selection

Results from a preliminary (ad hoc) search are provided in Supplementary Material 4. The systematic search will be conducted within MEDLINE (Ovid) and Embase (Ovid) from inception to 1st July 2023 (Supplementary Material 5). The search will be updated within a 12-month period closer to publication. Additionally, reference lists (backwards citation chasing) will be checked. Search results will be exported using EndNote software (EndNote X9, Clarivate, Philadelphia, USA, 2013) and imported into Covidence software (Covidence, Veritas Health Innovation, Melbourne, Australia) where duplicates will be removed. Two reviewers (P. K. and A. A.) will independently screen all unique studies first by title/abstract, followed by a review of full texts for those studies that appeared potentially relevant; disagreement is resolved by consensus discussion with a third reviewer (J. P.). Full-text review will be based on a checklist in which across five domains we will assess whether the study can be defined as target trial emulation study. The five domains for the checklist are prespecified as follows: indication, causal research question, prespecified target trial criteria, time 0, and emulation [5].

### Data items and data extraction

We will extract all the key variables listed in Table 2.

**Table 2:** Data items for data extraction

Variable	Definition	Variable type
DT	Date/time extraction	Date/time
Title	Title of the article	Text
Type	Type of article	Text
Name	Journal’s name	Text
Date	Date of publication (dd-mmm-yyyy)	Date
Authors	Complete list of authors	Text
Origin	Countries of origin	Text
DOI	Digital object identifier system	Text
Keywords	Keywords	Text
Source	Data source	Text
Aims	Aims of the study	Text
Number	Study population and sample size	Continuous numeric data
Study design	Type of study design	Text
Research framework	Population	Text
	Intervention	Text
	Comparator	Text
	Outcome	Text
Key findings	Key findings that relate to target trial emulation review questions	Text
Funding	Funding source	Text
Conflict of interests	Author’s conflicts are reported (yes/no), and description of conflicts of interests is provided	Text

#### Risk-of-bias (quality) assessment

The quality of reporting will be assessed using the RECORD-PE^4^ checklist, and the risk of bias will be based on a sophisticated adapted checklist by ROBINS-I^5^ and others^6^. To enable description of the characteristics and the quality of reporting of target trial emulation in each report, at least two reviewers (A. A., P. K., A. N.) will gather information from all studies using an adapted checklist which is based on a previously published data extraction form [5, 20, 21] (Supplementary Material 6). The extraction will be compared, and a third reviewer (J. R.) was included to resolve any differences. Where necessary (e.g., for design pre-specification criteria), the checklist (Supplementary Material 6) will be updated iteratively and data, supplements, and or protocols obtained from the investigators.

We aim to address three core questions using the information extracted from the prespecified data form (Supplementary Material 6). First, we will assess the design. The design is valid if the authors defined a specific target trial with an appropriate causal research question where general uncertainty exists, and patients could have received one or the other intervention. Second, we assess the emulation itself. The emulation is well conducted if the dataset is robust enough for the emulation and eligibility, and timepoint 0 and the start of follow-up are synchronized. Third, we will assess the robustness of the effect estimate. We will consider it to be robust if matching was done appropriately, biases were controlled for, measurable confounders accounted for, and sensitivity analyses performed and the interpretation of results in context of available evidence (Table 3).

**Table 3:** Adapted checklist for synchronization assessment [20]

Guiding question	Explanation
1. When does the follow-up start?	• Check if the authors report the start of follow-up. It might be called the baseline, index date, and time zero
2. When do individuals complete eligibility?	• Check if authors report when individuals should complete eligibility
2.a. Can individuals be eligible at multiple times?	• Check if individuals could be eligible at multiple times and whether authors used a strategy to overcome this: (1) choose a single eligible time, and (2) choose all eligible times and conduct a sequence of trials at each eligible time
2.b. Is there any post-baseline event (i.e., an event after the follow-up starts) in the eligibility criteria?	• Check if any events after the start of follow-up are listed in the eligibility criteria, e.g., complete two consecutive prescriptions or no outcome for the first 2 months after the start of follow-up
3. When are individuals assigned to an exposed or nonexposed group?	• Check if the authors report clearly when individuals are classified as exposed or nonexposed group
3.a. Do individuals have to use treatment for a given period to be classified as an exposed group?	• Check if individuals have to use treatment for a given period, e.g., complete two consecutive prescriptions to be classified as exposed and non-exposed, and if not, start the treatment or complete only 1 prescription
3.b. Is there a grace period?	• Check if individuals can start the treatment sometime after the start of follow-up and eligibility

### Strategies for data synthesis

The characteristics of all included studies and their risk-of-bias assessments will be compiled by at least two reviewers (A. A., P. K., A. N.) and checked by another author (J. P.) to resolve any differences. The findings will be grouped to summarize the scope of research questions addressed, methodological approaches used, and descriptive statistics will be provided for study characteristics. Risk-of-bias assessments will be grouped according to the domains of criteria such as eligibility, prespecified protocols, dataset quality, time-point synchronization, effect estimate reliability, and comparison to trial data. We will explore the differences between studies that qualified and failed in the use of the target trial approach to identify the introduction of bias. We will present which, why, and at what stage studies failed in applying the target trial approach and thus subverted the methodology. We will use specific examples, identifying the most common points of failure, the typical biases, and discuss how these could have been avoided if the methodology was applied correctly encountered in different settings such as drug trials, surgeries, and other interventions. Supplementary Material 7 summarizes the tables we plan to present our data with.

### *Meta*-*bias*(es)

There will be no assessment of publication bias, and the impact of publication bias in the field of trial emulation is currently unknown. In our review, we have specified clinical outcomes that are important for clinical decision-making. To assess reporting bias, we will review reference lists of included studies, appendices, and online registration databases to review study protocols and examine possible switching of primary outcomes. However, given there is no requirement for registration of trial emulation studies, comprehensive examination of reporting bias will be limited.

### Confidence in cumulative evidence

As this review will focus on the methodological conduct of target trial emulation in nephrology, no assessment of the certainty of the evidence will be undertaken.

## Discussion

The protocol outlines the conduct of a systematic methodological review that should provide guidance to better understand the validity and utility of the target trial framework in kidney failure. Our critical appraisal will compare studies that qualify for target trial emulation and provide reliable effect estimates against studies that have failed to do so by highlighting different potential biases. The overview will provide a strong rationale for clinical researchers and readers on how to properly apply the framework to enhance rigor and clarity in the provision of effect estimates that are not based on randomized trials. It will outline which biases and or errors should be avoided (and where possible how to avoid them) and facilitate conduct and reporting of sound observational studies attempting to evaluate treatment effect estimates.

Despite the comprehensive nature of the protocol that outlines this review, there are inherent limitations. The review may be limited by the availability and quality of the included studies, potential limited assessment of reporting and non-reporting bias as we will only include full-text publications, and heterogeneity in study designs and reporting standards. These limitations could impact the internal and external validity of our findings. Additionally, the reliance on existing literature means that some relevant studies might be missed due to language or publication status. However, by systematically addressing these issues and making recommendations for future research, we aim to mitigate these limitations and enhance the reliability of target trial emulation studies in kidney failure.

## Supplementary Information

Supplementary Material 1: Adapted PRISMA-P checklist.

Supplementary Material 2: Kidney disease outcome nomenclature.

Supplementary Material 3: Exclusion criteria.

Supplementary Material 4: Studies identified in a preliminary search.

Supplementary Material 5: Search strategy.

Supplementary Material 6: Adapted Appraisal Checklist [20, 22–24].

Supplementary Material 7.

## Data Availability

The corresponding author will make all data and materials available upon request.
