# Supplementary material for: Review of the target trial methodological approach on treatment effect estimates in kidney failure: protocol for a systematic assessment
Source: Syst Rev. 2024 Nov 14;13:280. doi: 10.1186/s13643-024-02672-4 (PMC11566441; doi:10.1186/s13643-024-02672-4)
Supplement: Supplementary file 2 — Supplementary Material 2: Kidney disease outcome nomenclature. [file 13643_2024_2672_MOESM2_ESM.docx]

**SUPPLEMENTARY MATERIAL 2: Kidney disease outcome nomenclature**

In an effort to attain better uniformity, the 2019 Kidney Disease: Improving Global Outcomes (KDIGO) Consensus Conference evaluated the terminology used to describe kidney disease. The suggestions were released in 2020, and it is anticipated that the new nomenclature would eventually replace the terminology that is already widely used. The words end-stage kidney disease (ESKD) or end-stage renal disease (ESRD) are more likely to be used in existing literature than the newly proposed terms kidney failure (KF) or end-stage renal disease (ESRD) in this review's summaries of findings. How these terms are used in this review is shown in the table below.

| **KDIGO 2020 nomenclature** | **Definition** | **Related terms** |
| --- | --- | --- |
| Kidney failure (KF) | GFR <15 ml/min per 1.73 m^2^ or treatment by dialysis  For ≥3 months | End-stage kidney disease (ESKD)  End-stage renal disease (ESRD)  End-stage kidney failure (ESKF)  End-stage renal failure (ESRF) |
| Kidney replacement therapy (KRT) | Includes dialysis and transplantation | Renal replacement therapy (RRT) |
| Kidney failure with replacement therapy (KFRT) | CKD G5 treated by dialysis or CKD G1-G5 after  transplantation; for epidemiologic studies, both should be  included | ESKD/ESRD/ESKF/ESRF requiring dialysis/transplantation |
| Kidney failure without replacement therapy (CKD G5 without KRT) | CKD G5 where KRT is not chosen or not available | End-stage kidney disease (ESKD)  End-stage renal disease (ESRD) |
| Chronic kidney disease without KRT (CKD without KRT) | CKD G1–G5, A1–A3 of any cause, not receiving dialysis or transplantation |  |
