# Supplementary material for: Review of the target trial methodological approach on treatment effect estimates in kidney failure: protocol for a systematic assessment
Source: Syst Rev. 2024 Nov 14;13:280. doi: 10.1186/s13643-024-02672-4 (PMC11566441; doi:10.1186/s13643-024-02672-4)
Supplement: Supplementary file 3 — Supplementary Material 3: Exclusion criteria. [file 13643_2024_2672_MOESM3_ESM.docx]

# SUPPLEMENTARY MATERIAL 3: EXCLUSION CRITERIA

Please select primary reason for exclusion.

Please note:

- A paper may be excluded for multiple reasons but just indicate one.
- Additional reasons may be identified.

Title, abstract and full-text review

- Genetics, obstetrics, prognostic, diagnostic, vaccine studies, etc.
- Clinical practice guideline
- Duplicate article
- Economic study (economic evaluation, cost-effectiveness)
- Randomized controlled trials
- Other epidemiological study (case-control, cross-sectional study, case series, case report)
- Excluded population (clinician, children, adolescents, community, caregivers)
- Non-therapeutic interventions: large scale public health interventions, diet, following guidelines, policy recommendations, QoL, survey, etc.
- Non-primary research (editorial, ethics, commentary, letter, news article, review, research protocols, policy documents)
- Survey study (Quantitative only)
- Studies with unsensible Endpoints
- Non-English/Non-German language article
- Studies not using target trial emulation
