# Supplementary material for: Review of the target trial methodological approach on treatment effect estimates in kidney failure: protocol for a systematic assessment
Source: Syst Rev. 2024 Nov 14;13:280. doi: 10.1186/s13643-024-02672-4 (PMC11566441; doi:10.1186/s13643-024-02672-4)
Supplement: Supplementary file 4 — Supplementary Material 4: Studies identified in a preliminary search. [file 13643_2024_2672_MOESM4_ESM.docx]

# SUPPLEMENTARY MATERIAL 4: STUDIES IDENTIFIED IN A PRELIMINARY SEARCH

| Study ID* and year | N* | Population (age, kidney disease) | Setting and Data Source | Methods | Research question (Intervention/Comparator) | Outcomes | Main finding |
| --- | --- | --- | --- | --- | --- | --- | --- |
| Fotheringham 2021* | 10 637 | 61.9 years, hemodialysis | UK, ARO (Analyzing Data, Recognizing Excellence and Optimizing Outcomes) cohort | Marginal structural cox proportional hazards models, logistic regression, inverse probability weighting | Survival on four compared with three times per week hemodialysis in high ultrafiltration patients | Mortality, hospitalisation, vascular access complications | No difference between four compared to three times per week hemodialysis |
| Fu 2021* | 4803 | 74 (IQR, 64-81) years, advanced chronic kidney disease | Swedish Renal Registry (2007-2017) | Propensity score-weighted cox proportional hazards regression | Comparative effectiveness of renin-angiotensin-system inhibitors compared to calcium channel blockers in individuals with advanced chronic kidney disease | Initiation of renal replacement therapy (primary), all-cause mortality and major adverse cardiovascular events (secondary) | Initiation of renin-angiotensin system inhibitor therapy compared with calcium channel blockers confers renal benefits with similar cardiovascular protection |
| Fu, 2021* | 10254 | 72 (63-79) years, advanced chronic kidney disease | Swedish Renal Registry (2007-2017) | Per protocol analysis: effect of adhering to the treatment strategies by censoring step of cloning, censoring, weighting method | Comparing the effect of stopping vs. continuing Renin-Angiotensin System Inhibitors | 5-year all-cause mortality (primary), major adverse cardiovascular events and renal replacement therapy (secondary) | Compared with continuing RAS inhibition, stopping this therapy was associated with a higher absolute 5-year risk of death (40.9% versus 54.5%) and major adverse cardiovascular events (47.6% versus 59.5%), but with a lower risk of KRT |
| Fu, 2021* | 10290 | 73 (IQR, 63-80) years, advanced chronic kidney disease | Swedish Renal Registry (2007-2017) | censoring step of cloning, censoring, weighting method; dynamic marginal structural model | Early vs. Late Dialysis initiation comparing 15 different dialysis initiation strategies (eGFR 4-19 ml/min) | 5-year all-cause mortality (primary), major adverse cardiovascular events (secondary) | Very early initiation of dialysis was associated with a modest reduction in mortality and cardiovascular events |
| Kainz, 2022* | 2346 | 44 (+-14) years, on hemodialysis and waitlisted for second transplant | Austrian Dialysis and Transplant Registry and Eurotransplant | sequential Cox approach, logistic regression, stabilized inverse probability of treatment/censoring weights | Difference in mean survival and all-cause mortality hazard comparing “retransplant” versus “waitlisted” after graft loss | All-cause mortality | Second kidney transplant is associated with patient survival compared with remaining waitlisted and treatment by dialysis, but the survival difference diminishes with longer waiting time. |
| Lyu, 2022* | 19867 | 77 (6,55) years, hemodialysis | US renal data system | High-dimensional propensity score and inverse probability of treatment weighting, and instrumental variable analysis | to compare arteriovenous fistula (AVF) versus arteriovenous graft (AVG) creation | All-cause mortality, all-cause and cause-specific hospitalization, and sepsis | No differences between AVGs and AVFs with respect to mortality, sepsis, or all-cause, cardiovascular-related, and infection-related hospitalization |
| McDermott, 2022* | 506 | 61 (18) years, e-GFR: 38.8 (IQR, 14- 72) | Mass General Brigham AAV cohort for patient identification  Outcome data linkage:  medical records, the US Renal Data System and the National Death Index | step of cloning, censoring, weighting method by inverse probability of censoring; Pooled logistic regression | effect of achieving a negative post induction anti-neutrophil cytoplasmic antibody ANCA) assay-  comparing patients who achieved versus did not achieve serological remission (negative ANCA assay) within 180 days of induction | Relapse, end-stage kidney disease or death within 5 years | Achieving serological remission within 180 days of induction was associated with lower risk of relapse, but no statistically significant difference in ESRD or mortality outcomes. |
| Shin, 2022* | 947900 | 60 (12) years, e-GFR 81.1 (6.17) ml/min | OptumLabs Data Warehouse | inverse probability of treatment–weighted hazard ratios (HRs) | Association of Rosuvastatin Use with Risk of Hematuria and Proteinuria | Hematuria, proteinuria, and kidney failure with replacement therapy | rosuvastatin was associated with increased risk |
| Strohmaier, 2022 (Austrian National Bank) | 4445 | 52.2 [13.2] years, hemodialysis | Austrian Dialysis and Transplant Registry and Eurotransplant | Sequential Cox approach | To compare restricted mean survival times (RMSTs) between patients who underwent transplant and patients continuing dialysis across transplant candidate ages and depending on waiting time | time from transplant allocation to death | Transplant was associated with increased survival time across all considered ages compared with continuing dialysis and remaining on the wait list within a 10-year follow-up |
| Wei, 2022* | 10554 | 40 to 89 years, moderate-to-severe chronic kidney disease plus gout | The Health Improvement Network U.K. primary care database (2000 to 2019) | Propensity score matching, cloning, censoring, and weighting approach, cox proportional hazard models | to assess the relation of allopurinol initiation to mortality and to examine the effects of achieving target serum urate level with allopurinol and allopurinol dose escalation on mortality. | 5-year all-cause mortality | Allopurinol initiation was associated with a modestly lower mortality compared with non-allopurinol use.  a treat-to-target approach of lowering SU level with allopurinol does not seem to increase mortality |
| Xie, 2020* | 216558 | 65.46 (11.05) years, type2 diabetes mellitus with mild chronic kidney disease | VA Health Care System | Propensity scores, logistic regression, inverse probability weighting | Comparative effectiveness of sodium–glucose cotransporter 2 inhibitors (SGLT2i), glucagon-like peptide 1 receptor agonists (GLP-1), dipeptidyl peptidase 4 inhibitors (DPP-4), and sulfonylureas | Composite outcome of estimated glomerular filtration rate (eGFR) decline >50%, end-stage kidney disease (ESKD), or all-cause mortality | Compared with those treated with sulfonylureas, treatment with SGLT2i, GLP-1, and DPP-4 was associated with a lower risk of the composite outcome |

**Abbreviations: Age,** mean (SD); unless otherwise specified**.** e-GFR = estimated glomerular filtration rate. *=publicly funded
