# Supplementary material for: Review of the target trial methodological approach on treatment effect estimates in kidney failure: protocol for a systematic assessment
Source: Syst Rev. 2024 Nov 14;13:280. doi: 10.1186/s13643-024-02672-4 (PMC11566441; doi:10.1186/s13643-024-02672-4)
Supplement: Supplementary file 5 — Supplementary Material 5: Search strategy. [file 13643_2024_2672_MOESM5_ESM.docx]

# SUPPLEMENTARY MATERIAL 5: SEARCH STRATEGY

Embase 1974 to present

1 exp renal replacement therapy/

2 kidney disease/

3 chronic kidney disease/

4 kidney failure/

5 chronic kidney failure/

6 mild renal impairment/

7 stage 1 kidney disease/

8 moderate renal impairment/

9 severe renal impairment/

10 end stage renal disease/

11 renal replacement therapy-dependent renal disease/

12 diabetic nephropathy/

13 kidney transplantation/

14 renovascular hypertension/

15 (hemodialysis or haemodialysis).tw.

16 (hemofiltration or haemofiltration).tw.

17 (hemodiafiltration or haemodiafiltration).tw.

18 dialysis.tw.

19 (CAPD or CCPD or APD).tw.

20 (kidney disease* or renal disease* or kidney failure or renal failure).tw.

21 (CKF or CKD or CRF or CRD).tw.

22 (ESRF or ESKF or ESRD or ESKD).tw.

23 (predialysis or pre-dialysis).tw.

24 ((kidney or renal) adj (transplant* or graft* or allograft*)).tw.

25 1 or 2 or 3 or 4 or 5 or 6 or 7 or 8 or 9 or 10 or 11 or 12 or 13 or 14 or 15 or 16 or 17 or 18 or 19 or 20 or 21 or 22 or 23 or 24

26 compar* effectiveness.tw.

27 causal inference.tw.

28 ((target adj2 trial) or (hypothetical adj trial) or (target adj2 emulation) or emula*).tw.

29 Inverse probability*.tw.

30 clone*, censor*,weight*.tw.

31 26 or 27 or 28 or 29 or 30

32 25 and 31

Medline (Ovid MEDLINE® Epub Ahead of Print, In-Process & Other Non-Indexed Citations, Ovid MEDLINE® Daily and Ovid MEDLINE®) 1946 to present - ScoR

1 Kidney Diseases/

2 exp Renal Replacement Therapy/

3 Renal Insufficiency/

4 exp Renal Insufficiency, Chronic/

5 Diabetic Nephropathies/

6 diabetic nephropath*.tw.

7 exp Hypertension, Renal/

8 dialysis.tw.

9 (hemodialysis or haemodialysis).tw.

10 (hemofiltration or haemofiltration).tw.

11 (hemodiafiltration or haemodiafiltration).tw.

12 (kidney disease* or renal disease* or kidney failure or renal failure).tw.

13 (ESRF or ESKF or ESRD or ESKD).tw.

14 (CKF or CKD or CRF or CRD).tw.

15 (CAPD or CCPD or APD).tw.

16 (predialysis or pre-dialysis).tw.

17 Uremia/

18 (ur?emic or ur?emia).tw. 33264

19 1 or 2 or 3 or 4 or 5 or 6 or 7 or 8 or 9 or 10 or 11 or 12 or 13 or 14 or 15 or 16 or 17 or 18

20 compar* effectiveness.tw.

21 causal inference.tw.

22 ((target adj2 trial) or (hypothetical adj trial) or (target adj2 emulation) or emula*).tw.

23 Inverse probability*.tw.

24 clone*, censor*,weight*.tw.

25 20 or 21 or 22 or 23 or 24

26 19 and 25
