# Supplementary material for: Review of the target trial methodological approach on treatment effect estimates in kidney failure: protocol for a systematic assessment
Source: Syst Rev. 2024 Nov 14;13:280. doi: 10.1186/s13643-024-02672-4 (PMC11566441; doi:10.1186/s13643-024-02672-4)
Supplement: Supplementary file 6 — Supplementary Material 6: Adapted Appraisal Checklist [20, 22–24]. [file 13643_2024_2672_MOESM6_ESM.docx]

# SUPPLEMENTARY MATERIAL 6: ADAPTED APPRAISAL CHECKLIST [20, 22, 23]

1. *Study characteristics (free text)*

- Author, year, study name, DOI, journal name, study origin, funding source

1. *Population (y/n)*

- Chronic kidney disease, dialysis patients, transplant patients

1. *Indication (y/n)*

- Drug, intervention, transplantation

1. *Data type (y/n, free text)*

- Were electronic health records or electronic medical records used?
- If not, what type of data were used (cohort study data, registry, health administration, pharmacy, insurance)
- Specific name of observational database

1. *Reporting (y/n)*

- Did the authors follow a reporting guideline[24]?
- If yes which guideline?

1. *Research question (y/n, free text)*

- Was the objective of the study reported and the research question valid?
- What was the objective?

1. *Target trial prespecification*

*Design*

- Was the target trial emulation design reported? Did they explicitly design a target trial?
- Did they use a diagram to illustrate key aspects?

*Setting*

- How well the study setting described in regards to setting, location, time/period of data collection?

*Eligibility criteria*

- Was the eligibility criteria reported? What was the target population?
- Did they specify exclusion of patients due to contraindications?
- Did they specify if patients were included once or multiple times?
- Did they report the code or algorithm used to select patients?

*Treatment strategy*

- Did they describe the treatment strategy/strategies?
- How many treatments were compared?
- What treatments were compared?
- Did they specify the data source from which the treatment information was obtained?
- If YES, what was/were the data source(s)?
- If YES, how reliable is/are the data source(s)?
- Did they report how and when patients initiate treatment?
- Did they report a time window to initiate the intervention? (grace period)
- Did they specify the comparator(s)?
- If YES, what kind of comparator(s) was it?
- If ACTIVE COMPARATOR(S), was there a time window for initiation to consider?
- Did they outline an approach to handle more than one relevant drug exposure?

*Follow-up period*

- Did they prespecify the start of the follow-up?

*Outcome*

- Did they report the outcome of interest?
- How sensitive was the outcome to the intervention?
- Did the authors prioritize/restrict outcome definition for the primary analysis?
- Did they report the code and algorithm to classify the outcome?

*Treatment assignment procedures*

- Were treatment administered at one point in time or sustained over time?
- Was there a GRACE period? If yes, what was it?
- If there was a grace period, was there a risk of misclassification of treatment?
- If there was a risk of misclassifying the treatment, how was it addressed?
- Does treatment assignment depend on any prognostic factor? (time varying treatment)

- If YES, how was it addressed?

- Randomly assign the individual to one of the strategies
- Clone exact copies of individuals and assign each clone to one of strategies then censor clone when strategies are deviated from. Adjustments needed for post-time zero selection bias (e.g., via IP weighting).
- No solution described.

- Are the investigators interested in the effect of initiating a treatment or the effect of a sustained treatment?

If interested in the effect of a sustained treatment, did they account for time-varying confounders?

Which statistical method did the authors use to achieve treatment arm balance?

1. *Defining time zero*

- When does follow-up start for a patient (baseline)?
- When do patients initiate the treatment (treatment assignment)?
- When do patients meet the complete eligibility criteria?
- Was time zero, treatment assignment and completed eligibility synchronized? (Y/n)
- If NO, choose one of the scenarios below:

*T0= time zero (dotted line), E= eligibility criteria, A= treatment strategy

A) Time zero is set after eligibility and strategy assignment


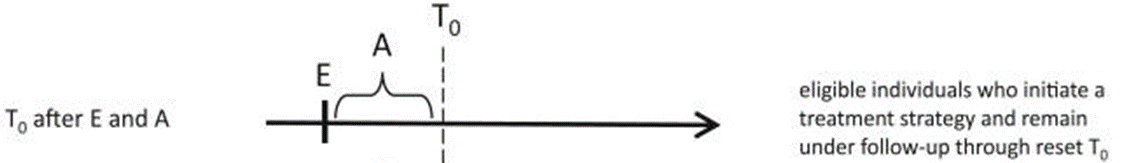


B) Time zero is set at eligibility but after strategy assignment


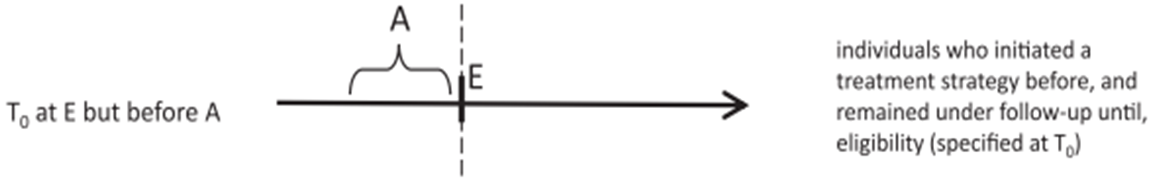


C) Time zero is set before eligibility


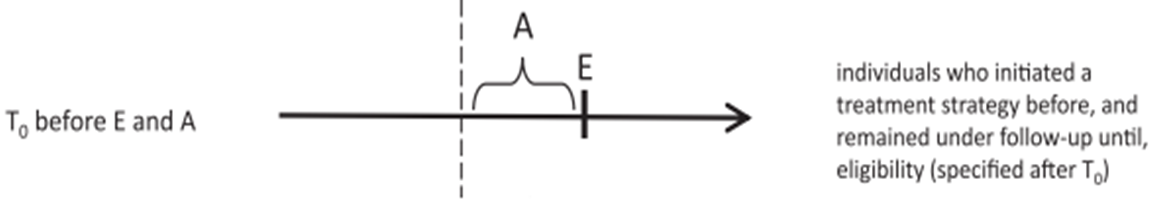


D) Time zero is set at eligibility, but treatment is assigned after time zero


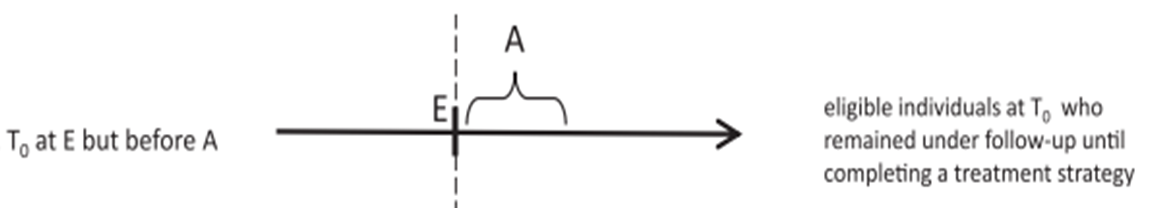


- If time zero, treatment assignment and completed eligibility were NOT synchronized, what bias was introduced?
- Selection bias
- Immortal time bias

- Did the authors have a strategy to address the bias introduced? (Y/N)
- If YES, what was the strategy for immortal time bias?
  - Avoided at design
  - Cloning technique?
  - Sequential trial emulations approach
- Was time zero hard to define? (Y/N) (i.e. Patients can have time zero at multiple time points.)
- If YES, how was it addressed?
- Choose one of the multiple times as time zero (the first time zero or a randomly chosen one)
- Choose all eligibility times as time zero and conduct a sequence of nested trials
- Choose some of the eligible times as time zero and match person-time when initiation occurs with person-time when no initiation occurs.
- No solution described
- Was there a problem of treatment adherence? (Y/N)

- If YES, how was it addressed?

- Estimate the per-protocol effect by defining time zero at treatment assignment and censoring when patients deviate from assigned strategy. Adjustments needed for confounding at time zero and post-time zero censoring.
- No solution described

1. *Causal contrast and analysis plan*

- What was the estimand of interest?
- For the statistical analysis, did they use:
- Per-protocol analysis
- Intention-to-treat analysis
- Both
- What was the measurement scale of the outcome?
- Which effect size measure was used to quantify the causal contrast of interest?
- Which method was used for analyzing the primary outcome?
- How robust is the statistical method being used?
- Were sample size calculations provided?
- If yes, what was determined
- Were adjustments made for baseline confounding factors? (Y/n)
- . If Yes,
  - matching with propensity scores? (Y/n)
  - inverse probability weighting?
  - Or other multivariable approaches?
- Linear regression
- Stratification
- Standardization
- G-estimation
- Other:
- Has a sensitivity analysis been performed? (Y/n)
  - If YES, which is the primary?
- Was selection bias due to loss to follow-up addressed explicitly?
- If so, how were missing data handled (inverse probability of censoring weighting, multiple imputation)?

1. *Reporting: Discussion/Conclusion*

- Did the author discuss the limitations of the study? (Y/n)
- Does the conclusion include causal language (causal vs association)? (Y/N)
- Does the conclusion or discussion include any spin? (Y/n)
- Have authors suggested conducting future studies? (Y/n)
- If YES, what kind of study/studies?
- Randomized control trial
- Replication studies
- Other observational studies
- Further research
- Other:

Extra Notes/Comments:
