# Supplementary material for: Review of the target trial methodological approach on treatment effect estimates in kidney failure: protocol for a systematic assessment
Source: Syst Rev. 2024 Nov 14;13:280. doi: 10.1186/s13643-024-02672-4 (PMC11566441; doi:10.1186/s13643-024-02672-4)
Supplement: Supplementary file 7 — Supplementary Material 7. [file 13643_2024_2672_MOESM7_ESM.docx]

# SUPPLEMENTARY MATERIAL 7

| Table 1: Summary of baseline characteristics of included studies | | |
| --- | --- | --- |
| Item | References | Number of studies, % or No. |
| Population |  |  |
| Chronic kidney disease stage 4 or higher |  |  |
| Transplant patients |  |  |
| Dialysis patients |  |  |
| Number of patients, *mean, SD* |  |  |
| 1 till 999 |  |  |
| 1000 till 9999 |  |  |
| 10000 till 99999 |  |  |
| 100000 till 999999 |  |  |
| Type of Data source |  |  |
| National Registry |  |  |
| Electronic Health Record |  |  |
| Other |  |  |
| Data: Countries of origin |  |  |
| Europe |  |  |
| United States/ South Amerika |  |  |
| Asia/Pacific |  |  |
| Year of publication |  |  |
| Before 2020 |  |  |
| After 2020 |  |  |
| Type of Intervention |  |  |
| Pharmacological |  |  |
| Transplantation |  |  |
| Surgery |  |  |
| Other Interventional |  |  |
| Type of Outcome |  |  |
| Mortality |  |  |
| Cause Specific Mortality |  |  |
| Study specific composite |  |  |
| Other |  |  |
| Study design |  |  |
| Active comparator new user design |  |  |
| Sequential trial design |  |  |
| Clone censor weight design |  |  |
| /Impact Factor of peer-reviewed publication |  |  |
| < 05 |  |  |
| < 10 |  |  |
| > 10 |  |  |
| Specification of target trial emulation |  |  |
| Abstract |  |  |
| Body |  |  |
| None |  |  |
| Funding |  |  |
| Non-profit |  |  |
| Profit |  |  |
| Both |  |  |
| None |  |  |
| Statistical Methods |  |  |
| Inverse probability treatment weighting |  |  |
| Matching with propensity score |  |  |
| Matching without propensity score |  |  |

# Legend table 1: %= Percentage, SD= standard deviation

| Table 2: Studies qualifying for target trial emulation | | | | | | | |
| --- | --- | --- | --- | --- | --- | --- | --- |
| Author, yr | **Target trial definition ^1^** | **Type of research question ^2^** | **Prespecified analysis plan or protocol** | **Datasource robust enough for emulation ^3^** | **Synchronisation** | **Reliable effect estimate** | **Comparison to trial data** |
| *Assimon, 2018^4^* | *Association of carvedilol versus metoprolol initiation and 1-year all-cause and cardiovascular mortality* | *Comparative Effectiveness* | *Detailed and prespecified analysis plan* | *US Renal Data System linked with Electronic Health Record of US dialysis provider (DaVita)* | *Washout period before beta blocker initiation* | *Control of biases, Adjustment of confounders, Sensitivity analysis* | *Comparison to trial data unavailable but reference to broader literature* |

Table 2 legend: Explanation: 1: Causal question and patient-level intervention equipoise 2: Efficacy, Comparative effectiveness, Safety; 3: Data source robustness:

large representative cohort.

Citation: 4 Assimon MM, Brookhart MA, Fine JP, Heiss G, Layton JB, Flythe JE. A Comparative Study of Carvedilol Versus Metoprolol Initiation and 1-Year Mortality Among Individuals Receiving Maintenance Hemodialysis. Am J Kidney Dis. 2018 Sep;72(3):337-348. doi: 10.1053/j.ajkd.2018.02.350. Epub 2018 Apr 10. PMID: 29653770; PMCID: PMC6477681.

| Table 3: Reasons for failing target trial emulation | | | | | | | |
| --- | --- | --- | --- | --- | --- | --- | --- |
| Author, yr | **Research question** | **Design Features** | **Eligibility** | **Synchronization of time point 0** | **Dataset** | **Stat. execution** | **Other features** |
| *Avni-Nachman, 2021^7^* | *Fulfilled: Compare short (6-10 days) versus prolonged (11-21 days) antibiotic treatment for compl. UTI among kidney transplant recipients* | *Not Fulfilled:*  *Reason: Design not prespecified (results include unexplained analysis)* | *No Equipoise: Patients not eligible for both arms*  *Reason: You cannot allow Important prognostic factors to be different such as, time since transplant, blood stream infection)* | *Partially fulfilled: Reason: Time point 0 the same in both groups but immortal time bias introduced (people died between day 6 and day 10 in short treatment group)* | *Fulfilled* | *Partially Fulfilled:*  *Reason: Not intention-to-treat; Compared patients to antibiotic therapy they actually got and not their initial prescription; Inverse probability weighting does not fix design errors in ineligibility* | *Not enough outcome events to enable reliable estimates* |

Table 3 legend. Explanation and or Abbreviation: ^1^ No causal question, i.e. exposure or risk factor related. ^2^ Error in choice of PICO criteria*. ^3^Ineligible to receive either treatment. ^4^* Synchronization of timepoint 0, eligibility and start of specified follow-up. *^5^* Does the dataset contain the necessary information to ascertain intervention and outcome. ^6^ Way of matching; sufficient control of biases and adjustment of confounders (y/n)
Citation: 7 Avni-Nachman S, Yahav D, Nesher E, Rozen-Zvi B, Rahamimov R, Mor E, Ben-Zvi H, Milo Y, Atamna A, Green H. Short versus prolonged antibiotic treatment for complicated urinary tract infection after kidney transplantation. Transpl Int. 2021 Dec;34(12):2686-2695. doi: 10.1111/tri.14144. Epub 2021 Nov 9. PMID: 34668610.

| Table 4: Most common biases | | |
| --- | --- | --- |
| Type of bias | **References** | **Number of studies or %** |
| Selection bias^1^ |  |  |
| Immortal time bias^2^ |  |  |
| Lead time bias^3^ |  |  |

Table 4 legend: 1 Selection bias in target trial emulation occurs when the participants in an observational study are not representative of the population that would have been in a randomized trial, often due to factors related to treatment decisions and outcomes. This bias can lead to distorted estimates of treatment effects.

2 Immortal time bias occurs when a period of follow-up during which the outcome cannot occur (immortal time) is incorrectly included in the treatment group, leading to an overestimation of treatment effects. This bias arises because individuals must survive through the “immortal time” to receive treatment, making the treatment appear more beneficial than it truly is.

3 Lead time bias occurs when earlier detection of a disease (e.g., through screening) falsely appears to improve survival time, even if the disease’s progression or outcome isn’t actually altered. This bias can give the impression of extended survival without changing the natural course of the illness.
